# Factors affecting attitudes toward colorectal cancer screening in the primary care population

**DOI:** 10.1038/sj.bjc.6605130

**Published:** 2009-06-23

**Authors:** T Taskila, S Wilson, S Damery, A Roalfe, V Redman, T Ismail, R Hobbs

**Affiliations:** 1 Primary Care Clinical Sciences, School of Health and Population Sciences, University of Birmingham, Edgbaston, Birmingham B15 2TT, UK; 2 Queen Elizabeth Hospital, University Hospital Birmingham Foundation NHS Trust, Birmingham B15 2TT, UK

**Keywords:** colorectal cancer, screening, attitude, ethnic minority, symptom

## Abstract

**Background:**

Colorectal cancer (CRC) is a major cause of death in the United Kingdom. Regular screening could significantly reduce CRC-related morbidity and mortality. However, screening programmes in the United Kingdom have to date seen uptake rates of less than 60%. Attitudes towards screening are the primary factors determining patient uptake.

**Methods:**

A questionnaire was sent to people aged 50–69 years who were registered with general practices in the West Midlands. A total of 11 355 people (53%) completed the questionnaire. Multivariable logistic regression analyses were performed to identify those factors (gender, age, ethnicity, deprivation, number of symptoms, and their duration) that most strongly contributed to negative/positive attitudes in the primary care population.

**Results:**

Fourteen percent of respondents had a negative attitude towards screening. Men, older people, and those with Indian ethnic backgrounds were more likely to have negative attitudes toward screening, whereas people with Black-Caribbean ethnic background, people with multiple symptoms and those reporting abdominal pain, bleeding, and tiredness were more likely to have a positive attitude.

**Conclusion:**

Culturally relevant screening strategies should aim to increase knowledge of the symptoms and signs related to bowel cancer among South Asian ethnic groups in the United Kingdom. It is also important to find ways to increase the acceptability of screening among asymptomatic patients.

Colorectal cancer (CRC) is the third most common cancer in the United Kingdom, and has the second highest cancer mortality rate, with over 15 000 deaths each year (Office of National Statistics, 2003). Around one in 20 people in the United Kingdom will develop CRC during their lifetime (Department of Health, 2000). Survival is inversely related to cancer stage, and up to 90% of CRC deaths may be preventable with early detection (Smith *et al*, 2001). However, most cases are currently diagnosed at a late stage, which is associated with a 5-year overall survival rate of only 48% (Coleman *et al*, 2008).

Colorectal cancer incurs an annual expenditure of more than £300 million in surgical, adjuvant, and palliative treatment (Macafee *et al*, 2006), which could be significantly reduced by earlier diagnosis and additionally, screening programmes could significantly reduce CRC morbidity and mortality.

The National Bowel Cancer Screening Programme was introduced in England in 2006, with coverage across the whole of the United Kingdom planned by 2010 (Atkin, 2006). The programme aims to screen men and women aged 60–69 years for CRC by using the Fecal Occult Blood test (FOBt). Pilot evaluations, in both Scotland and Rugby, confirmed the feasibility of a national screening programme; however, uptake rates were only 58.5% (UK Colorectal Cancer Screening Pilot Group, 2004) and 52% (Weller *et al*, 2007) in the first and second rounds of screening, respectively.

Low uptake rates of cancer screening have been found to be associated with socioeconomic status (McCaffery *et al*, 2002; Wardle *et al*, 2004), ethnic origin (Szczepura *et al*, 2003; Jerant *et al*, 2008; Johnson *et al*, 2008; Robb *et al*, 2008a), age and gender (Subramanian *et al*, 2004; Weller *et al*, 2007). Psychosocial factors, such as embarrassment, fear of cancer, and lack of knowledge, could also play a role in poor uptake (Subramanian *et al*, 2004; Smith *et al*, 2005). A positive attitude towards screening may be an important factor affecting screening attendance. A review of 44 studies examining factors affecting adherence with bowel cancer screening guidelines, found that a positive attitude towards screening was the primary factor correlated with patient uptake (Subramanian *et al*, 2004).

Little is known about the factors that affect attitudes toward CRC screening. Previous studies have been based on the existing data from registries of people who have participated in CRC screening. Our community-based study provides important information about current attitudes toward CRC screening among the population targeted by the UK National Bowel Cancer Screening Programme, and reports findings of direct relevance to the wider rollout of the programme across the United Kingdom. In addition to looking at the impact of gender and ethnicity, we were able to examine the effect of presence of symptoms on attitudes toward CRC screening. Investigating the relative influence of these factors allows greater understanding of both the predictors of poor uptake of screening, and the reasons that symptomatic people may have for not presenting for treatment.

Identifying the characteristics associated with the negative attitudes toward screening could allow the development of suitable education programmes which can be specifically targeted towards those who may be most reluctant to undergo CRC screening, thereby increasing its acceptability among these groups. The aim of this study was therefore to identify which factors (gender, age, ethnicity, deprivation, number of symptoms and their duration) most strongly contribute to negative attitudes to, and subsequently low uptake of, CRC screening in the primary care population.

## Materials and methods

### Study population

The methods are described in detail elsewhere (Wilson *et al*, 2006). In summary, people between 50 and 69 years of age, who were registered in one of 19 participating general practices in the West Midlands region of the United Kingdom were sent a survey asking about their gender, age, ethnic background, the presence of bowel-related symptoms during the past 3 months, duration of symptoms, and the perceived acceptability of screening for CRC. Those who were under investigation or treatment for CRC, unfit for colonoscopy, unable to give informed consent or who were unsuitable to participate for any other reason were excluded. A questionnaire was sent to 21 488 people, with one reminder sent to non-responders after 2 weeks. A total of 133 patients were excluded after mailing: 27 were deceased, 236 questionnaires were returned as undeliverable, 2402 people returned a blank questionnaire, and 7335 did not respond.

### Acceptability of CRC screening

Participants were asked about their attitudes toward three bowel screening tests: colonoscopy, FOBt, and flexible sigmoidoscopy (FS). The procedure was briefly outlined, and participants were asked to indicate their perceived acceptability of each test on a scale of 1 (very acceptable) to 5 (very unacceptable). Respondents' attitudes toward CRC screening were assessed by the following question: ‘Screening using the above procedures can be used to look for evidence of early bowel cancer and conditions that may progress to bowel cancer in people who have symptoms. Do you think this is a good idea?’ Responses were dichotomised between those who answered ‘yes’ (positive attitude to screening) and those who answered either ‘no’ or ‘not sure’ (negative attitude to screening).

### Symptoms related to CRC

Recording of symptoms was based on the Department of Health (DH) referral guidelines for suspected CRC (National Institute for Health and Clinical Excellence, 2005). Participants were asked whether they had had any of the following symptoms during the past 3 months: abdominal pain; weight loss; tiredness or weakness; blood in stools; bleeding from the back passage; change in bowel habit to harder stools; change in bowel habit to looser stools; change in bowel habit to needing to open the bowels less often than usual; change in bowel habit to needing to open the bowels more often than usual, and pain, soreness, discomfort, itching or lumps around the back passage. Participants also reported the duration of each symptom measured in weeks.

Respondents were allocated into one of the three categories: those who did not report any of the symptoms, those who had one or two symptoms, and those who had three or more symptoms.

### The index of multiple deprivation (IMD 2004)

The IMD2004 was used as a proxy measure of multiple deprivation. This index is a weighted area level aggregation of a number of distinct ‘domains’ of deprivation (income; employment; health inequality; disability, education, skills and training; barriers to housing services; crime, and the living environment). People may be counted as being in one or more of these domains, depending on the number and types of deprivation they experience (Incidents of Deprivation, 2004). Lower IMD scores indicate less deprived residential areas, whereas higher scores are associated with more deprived locations. Ranked data were converted to quartiles for analysis, with quartile 1 representing the most affluent group and quartile 4 the most deprived.

### Statistical analysis

Analysis of variance (ANOVA) and *χ*^2^ tests were performed to examine differences between ethnic groups according to other demographic and symptom-related variables. Logistic regression analysis was used to examine the relationship between negative attitudes to screening and the presence and duration of particular symptoms. The linearity assumption was examined for duration using a smoothed lowess plot of the logit along with fractional polynomial model comparisons.

Multivariable logistic regression analysis was performed to identify which variables had an independent effect on negative attitude towards screening after adjustment for all other variables in the model. Variables entered into the analysis included age group, gender, ethnic group, IMD2004 quartile, symptoms and/or duration of symptoms (identified from the earlier analyses of symptoms). A final parsimonious model was found using the backward elimination method. The statistical analyses were performed using STATA 9 (Version 9, StataCorp LP, College Station, TX, USA) and SPSS 14 (Version 14, SPSS Inc., Chicago, USA).

## Results

A total of 11 355 completed questionnaires were received (53%) and these respondents comprised the study population. Fifty-two percent of respondents were women and the mean age was 59.5 years. Non-responders were more likely to live in the most deprived areas than responders (46 *vs* 35%, *P*<0.0001). No other statistically significant differences between responders and non-responders were found.

People with white backgrounds were compared against those with other ethnic backgrounds on the basis of demographic characteristics (Table 1). A significantly greater number of respondents were female among the Black-Caribbean ethnic group than for the white ethnic group (65 *vs* 53%, *P*=0.008). A significantly younger age profile was found among respondents with an Indian ethnic background than among those with a white background (43 *vs* 25%, *P*<0.001). In terms of deprivation, respondents from both Black-Caribbean and Black-African ethnic groups were significantly more likely to live in the more deprived residential quartiles than people with white background (64 and 53 *vs* 34%, *P*<0.001 and 0.007, respectively).

The number of symptoms according to ethnic group is outlined in Table 2. Pakistani/Bangladeshi respondents were significantly more likely to report 1–2 symptoms related to CRC than those with a white ethnic background (49 *vs* 23%, *P*=0.03). Female respondents reported 3 or more symptoms more often than men (59 *vs* 41%, *P*<0.001). Tiredness was the most common symptom, reported by 20% of respondents; the mean duration of tiredness was 3.10 weeks. Those from the Black-Caribbean ethnic group reported ‘tiredness’ (30 *vs* 20%, *P*=0.004), ‘change in bowel habit to harder stools’ (20 *vs* 10%, *P*<0.001), ‘abdominal pain’ (16 *vs* 9%, *P*=0.004), and ‘weight loss’ (4 *vs* 2%, *P*=0.012) significantly more than those with a white ethnic background.

A total of 1458 responders (13% of the study population) found FOBt either *very unacceptable* or *unacceptable*. The corresponding percentages for colonoscopy and the FS were 2668 (23%) and 2518 (22%), respectively. A total of 1543 respondents (14%) were found to have a negative attitude towards CRC screening. Logistic regression analysis was undertaken to investigate whether attitudes toward screening were associated with any particular symptom or symptom duration. Univariate analyses indicated that the presence of each of the 10 symptoms were significant predictors of attitudes toward CRC screening. After controlling for age and gender; four symptoms remained as significant predictors of attitude, ‘tiredness’, ‘abdominal pain’, ‘bleeding from the back passage’, and ‘pain, soreness, discomfort, itching, or lumps around the back passage’. These symptoms were included in the final model. With the exception of tiredness, negative attitudes were best described by the symptom presence alone that is, there was no additional benefit of including duration of symptoms in the model. A lowess plot suggested a nonlinear relationship between the log odds of disease and duration of tiredness; it also showed considerable variability in the logit across the duration of tiredness symptoms. However, comparison of fractional polynomials identified the linear model as the model of best fit.

The final multivariable logistic model (Table 3) suggested that men (OR: 1.66; CI 1.47–1.88) and those over 65 years of age (OR: 1.23; CI 1.03–1.47) were more likely to have a negative attitude towards CRC screening. People from an Indian ethnic background were more likely to have a negative attitude than those from a white ethnic background (OR: 1.70; CI 1.18–2.46). In turn, participants reporting three or more symptoms (OR: 0.62; CI 0.38–0.92), and those with a Black-Caribbean background were more likely to have a positive attitude to screening (OR: 0.58; CI 0.34–0.98). Furthermore, the presence of abdominal pain, bleeding, tiredness, and duration of tiredness were associated with a positive attitude.

## Discussion

A total of 14% of the study population (*n*=1543) had a negative attitude towards CRC screening. Men, older people, and those with South-Asian ethnic backgrounds were more likely to have negative attitudes; whereas Black-Caribbean people, those with multiple symptoms, and those with abdominal pain, bleeding, and tiredness were more likely to have a compliant attitude.

Our findings are consistent with the results of the UK CRC screening pilot study, indicating a negative attitude towards CRC screening among men and people in older age groups (Weller *et al*, 2007). The influence of age was previously observed in the survey of opportunistically supplied FOBt kits by GPs, with 62% of patients aged 50–69 years returning kits compared with 54% aged 70 years or over (Hobbs *et al*, 1992). People over the age of 70 years tend to perceive themselves as having a lower risk of CRC (Robb *et al*, 2004), which could go some way towards explaining their less favourable attitude toward CRC screening.

In our study, females reported multiple symptoms significantly more frequently than male respondents. Nearly 60% of those who reported three or more symptoms were female; this could partly explain their more favourable attitude towards screening. On the other hand, the findings of the impact of gender on the uptake of bowel cancer screening have been equivocal. One recent systematic review of adherence to CRC screening found that women were less likely to comply (Subramanian *et al*, 2004), whereas another found no difference in terms of age and gender in referral delay (Macdonald *et al*, 2006). In our study, older age could not explain differences in attitudes between ethnic groups, as there were no significant differences in age distribution between the ethnic groups, except among those with an Indian ethnic background who were significantly younger than those with a white ethnic background. A UK CRC screening pilot report of the impact of ethnicity on screening uptake suggests that low uptake rates cannot be explained by differences in age, gender, or deprivation index (Szczepura *et al*, 2003). Similar findings have been reported elsewhere: in a recent study conducted in the USA, ethnic minority groups reported lower CRC screening attendance than people with white ethnic backgrounds. The results remained significant after controlling for demographic factors (Jerant *et al*, 2008). In our study, ethnicity was the strongest predictor of attitudes toward CRC screening after controlling for other factors, suggesting that negative attitudes toward screening are likely to be culturally influenced.

Our findings on the impact of ethnicity are in line with the results of the UK CRC screening pilot study, where a lower adherence for screening was reported among South-Asians compared with white and black participants (Szczepura *et al*, 2003). Black participants also showed strongest intentions to attend for screening, which reflects a positive attitude towards CRC screening among the members of this ethnic group (Robb *et al*, 2008a). In our study, Black-Caribbean respondents had a more favourable attitude towards CRC screening than white respondents. Considerably, more Caribbean respondents were women (65%), which could partly explain their more favourable attitude towards CRC screening. Some ethnic minority groups have also been found to be more likely to express fatalistic beliefs about cancer than people with white backgrounds (Subramanian *et al*, 2004; Johnson *et al*, 2008; Robb *et al*, 2008a). A belief about ‘tempting fate’ by undergoing cancer screening may therefore contribute to a negative attitude among some ethnic minority patients.

In our study, people with symptoms were more likely to have a positive attitude towards screening. People with multiple symptoms are also likely to perceive their risk of bowel cancer to be higher (Robb *et al*, 2004) and are less likely to delay referral (Mitchell *et al*, 2008). Patients tend to consult more quickly when their symptoms are, or are perceived to be, more serious, including the presence of pain or bleeding (Smith *et al*, 2005; Macdonald *et al*, 2006; Mitchell *et al*, 2008). We found that people with abdominal pain, bleeding from the back passage, and tiredness were more likely to have a positive attitude to CRC screening. We have demonstrated that 38% of this community sample were experiencing symptoms and 88% of these had a positive attitude toward screening. If, on receipt of an invitation for bowel screening, those who have symptoms come forward for investigation, then screening may facilitate earlier diagnosis. Nevertheless, the role of screening is to identify asymptomatic disease. Interventions are required to ensure that those with symptoms present promptly for investigation.

The attribution of illness often arises when symptoms develop and begin physically restricting everyday life, especially among men (Smith *et al*, 2005; Mastalski *et al*, 2008). In our study, with the exception of tiredness, duration of symptoms was not associated with reported attitudes toward CRC screening.

In terms of early referral, recognition of symptom seriousness may be more important than recognition of the presence of the symptom (Macdonald *et al*, 2006). Perception of symptom seriousness is often based on a personal or family history of similar symptoms (Smith *et al*, 2005; Macdonald *et al*, 2006; Mitchell *et al*, 2008). Family history has also been found to be a motivating factor for CRC screening attendance (Mastalski *et al*, 2008). A lack of knowledge of cancer symptoms has been found to be one of the main factors influencing referral delay (Mitchell *et al*, 2008).

South-Asians have been reported as being less aware of bowel cancer and less confident about the effectiveness of screening (Robb *et al*, 2008b). According to a population-based study, non-white respondents viewed their risk of bowel cancer as lower than their peers (Robb *et al*, 2004). There are ethnic differences in the risk of developing bowel cancer (Swerdlow *et al*, 1995; Renehan *et al*, 2008). People from lower-risk groups are therefore less likely to have direct experience of the disease among their family and friends; and are therefore less aware of the symptoms and signs of CRC. This could explain negative attitudes to CRC screening among some of the ethnic minority groups in our study.

## Limitations

One limitation of this study was the relatively low response rate of 53%. There was no non-response bias in terms of age or gender. There was, however, a significant difference on the basis of deprivation, indicating that those living in more deprived areas were significantly less likely to respond than those living in less deprived areas. Despite this, deprivation was not found to be a significant predictor of attitudes, suggesting that selection bias has not affected the validity of our findings. In addition, it is likely that one reason for non-response may have been a lack of interest in, or a negative attitude to, screening. This suggests that if there is any bias in our findings, it is that we have under-estimated the strength of factors such as ethnicity in influencing attitudes toward CRC screening.

A further limitation of this study is that a high percentage of respondents did not provide information on symptom duration; therefore, these effects could not be reliably measured. This could partly explain why the duration of symptoms was not found to be a significant determinant of attitudes toward CRC screening.

Finally, our analysis focused on the use of a single, dichotomised outcome measure in assessing respondents' attitudes toward CRC screening. If successful interventions are to be designed and targeted towards specific population subgroups to increase screening uptake, further research is needed to investigate in detail the determinants of attitudes reported by the members of these subgroups.

## Conclusion

To our knowledge, this is the first study examining attitudes toward CRC screening in the UK population being targeted for screening. Our results clearly show that gender, age, ethnicity, number of symptoms, and the presence of certain symptoms are the strongest predictors of attitudes toward CRC screening, and presumably influence the desirability of undergoing screening for CRC. Culturally relevant screening strategies should be developed. Increasing knowledge of the symptoms and signs related to bowel cancer among ethnic minority groups in the United Kingdom is a priority. Health-care authorities should also investigate ways in which the importance of CRC screening can be emphasised to members of the target screening population in order to increase the acceptability of screening among asymptomatic patients.

## Figures and Tables

**Table 1:** Study population (*n*=11 355) by ethnic group

**Explanatory variables, *n* (%)**	**White, *n*=10 270**	**Indian, *n*=240**	**Pakistani/ Bangladeshi, *n*=45**	**Black- Caribbean, *n*=237**	**Black-African, *n*=47**	**Chinese, *n*=52**	**Mixed, *n*=174**
*Gender*							
Male 5320 (47)	4826 (47)	122 (49)	22 (49)	84 (35)	19 (40)	26 (50)	87 (50)
Female 6035 (53)	5444 (53)	118 (51)	23 (51)	153 (65)	28 (60)	26 (50)	87 (50)
							
*Age (years)*							
50–54 2891 (26)	2548 (25)	101 (43)	17 (38)	82 (35)	17 (37)	17 (33)	42 (25)
55–59 3127 (28)	2849 (28)	62 (26)	16 (36)	43 (19)	11 (24)	20 (39)	50 (29)
60–64 2861 (25)	2659 (26)	31 (13)	6 (13)	45 (19)	13 (28)	4 (8)	40 (23)
65–69 2476 (21)	2074 (20)	41 (17)	6 (13)	63 (27)	11 (5)	11 (21)	39 (23)
							
*IMD*							
Quartile 1 1047 (9) (least deprived)	999 (10)	13 (6)	4 (9)	6 (3)	0 (0)	4 (7)	12 (7)
Quartile 2 2146 (19)	1959 (19)	67 (27)	7 (16)	18 (8)	4 (9)	10 (19)	27 (16)
Quartile 3 4194 (37)	3819 (37)	89 (37)	10 (22)	60 (25)	18 (38)	19 (37)	64 (37)
Quartile 4 3961 (35) (most deprived)	3487 (34)	71 (30)	24 (53)	152 (64)	25 (53)	19 (37)	71 (41)
							
*Number of symptoms*							
None 6991 (62)	6353 (62)	150 (63)	14 (31)	133 (56)	28 (60)	28 (54)	107 (61)
One or two 2577 (23)	2321 (23)	50 (21)	22 (49)	52 (22)	9 (19)	16 (31)	40 (23)
Three or more 1787 (16)	1596 (16)	40 (17)	9 (20)	52 (22)	10 (21)	8 (15)	27 (16)

**Table 2:** Symptoms reported, by ethnic group and symptom duration

**Symptom, *n* (%)**	**White, *n*=10270**	**Indian, *n*=240**	**Pakistani/ Bangladeshi, *n*=45**	**Black- Caribbean, *n*=237**	**Black- African, *n*=47**	**Chinese, *n*=52**	**Mixed, *n*=174**	**Duration in weeks Mean (s.d.)**
Tiredness or weakness 2231 (20)	1966 (20)	56 (24)	14 (34)	67 (30)	10 (23)	13 (27)	39 (24)	3.10 (23.76)
Pain, soreness, discomfort, itching, or lumps around the back passage 2054 (18)	1879 (19)	36 (15)	13 (30)	36 (16)	10 (23)	9 (18)	26 (16)	4.91 (50.35)
Change in bowel habit to looser stools 1446 (13)	1320 (13)	26 (11)	6 (14)	29 (13)	4 (10)	4 (8)	20 (12)	2.16 (28.40)
Change in bowel habit to needing to open your bowels more often than usual 1190 (11)	1075 (11)	27 (11)	8 (19)	26 (12)	8 (19)	4 (8)	13 (8)	1.60 (21.98)
Change in bowel habit to harder stools 1107 (10)	970 (10)	25 (10)	4 (9)	46 (20)	6 (14)	3 (6)	24 (14)	1.33 (22.84)
Bleeding from the back passage 1045 (9)	949 (9)	25 (10)	9 (21)	21 (9)	0	6 (12)	19 (11)	1.74 (34.67)
Abdominal pain 967 (9)	870 (9)	27 (11)	5 (12)	36 (16)	7 (16)	7 (14)	15 (9)	1.31 (16.73)
Change in bowel habit to needing to open your bowels less often than usual 677 (6)	597 (6)	15 (6)	4 (9)	25 (11)	3 (7)	1 (2)	10 (6)	1.37 (31.57)
Blood in stools 397 (4)	337 (3)	15 (6)	3 (7)	14 (6)	2 (5)	4 (8)	8 (5)	0.62 (15.03)
Weight loss 190 (2)	152 (2)	8 (3)	1 (2)	10 (4)	4 (10)	8 (4)	3 (2)	0.23 (4.32)

**Table 3:** Odds ratios with 95% confidence intervals for the likelihood of a negative attitude towards colorectal cancer screening by explanatory variables

**Variable**	**OR (95% CI)^a^**	***P*-value**
*Age (years)*		
50–54	1.00	
55–59	0.98 (0.83–1.16)	0.78
60–64	1.05 (0.89–1.25)	0.55
65+	1.23 (1.03–1.47)	0.02
		
*Sex*		
Female	1.00	
Male	1.66 (1.47–1.88)	<0.001
		
*Ethnicity*		
White	1.00	
Indian	1.70 (1.18–2.46)	0.005
Pakistani/Bangladeshi	1.57 (0.99–2.47)	0.06
Black-Caribbean	0.58 (0.34–0.98)	0.05
Black-African	1.75 (0.77–3.98)	0.18
Chinese	1.23 (0.50–3.00)	0.66
Mixed	1.55 (0.52–4.68)	0.45
		
*IMD*		
Quartile 1 (least deprived)	1.00	
Quartile 2	1.02 (0.80–1.31)	0.86
Quartile 3	1.19 (0.96–1.49)	0.11
Quartile 4 (most deprived)	1.17 (0.94–1.47)	0.16
		
*Number of symptoms*		
None	1.00	
One or two	0.58 (0.44–0.73)	<0.001
Three or more	0.62 (0.38–0.92)	0.02
		
*Symptoms*		
Abdominal pain	0.64 (0.43–0.99)	0.05
Bleeding from the back passage	0.61 (0.42–0.94)	0.02
Pain, soreness, discomfort, itching, or lumps around the back passage	0.75 (0.57–1.06)	
Tiredness	0.53 (0.36–0.78)	<0.001
Tiredness frequency (weeks)	1.005 (1.002–1.008)	<0.001

a Each explanatory variable (age, sex, ethnicity, IMD, number of symptoms, symptoms) adjusted for other explanatory variables.
